# Surgical ciliated cyst of the mandible after orthognathic surgery: a case report with review of the literature

**DOI:** 10.1186/s40902-022-00356-4

**Published:** 2022-08-01

**Authors:** Sungbin Youn, Hyun Jun Oh, Hye-Jung Yoon, Byoung-Moo Seo

**Affiliations:** 1 Department of Oral and Maxillofacial Surgery, School of Dentistry, Seoul National University, Seoul, South Korea; 2 Oral Oncology Clinic, National Cancer Center, Goyang, South Korea; 3 Department of Oral Pathology, School of Dentistry, Seoul National University, Seoul, South Korea; 4 Department of Oral and Maxillofacial Surgery, School of Dentistry and Dental Research Institute, Seoul National University, Seoul, 03080 Korea

**Keywords:** Surgical ciliated cyst, Postoperative maxillary cyst, Implantation cyst, Orthognathic surgery

## Abstract

**Background:**

Surgical ciliated cysts, also known as postoperative maxillary cysts or implantation cysts, occur mainly in the posterior maxilla after radical maxillary sinus surgery; they rarely develop in the mandible. They are thought to occur when the sinonasal epithelium is infiltrated by a surgical instrument during surgery or as a result of transplantation of bone or cartilage with respiratory epithelium attached.

**Case presentation:**

We report a case in which a surgical ciliated cyst developed in the anterior part of the mandible, presumably as a result of bimaxillary orthognathic surgery and genioplasty performed 24 years earlier. We then review the few similar cases reported in the literature.

**Conclusion:**

Surgical ciliated cysts in the mandible are extremely rare, but they could occur after simultaneous surgery on the maxilla and mandible, even decades later. To prevent surgical ciliated cysts in the mandible, we recommend that the surgical instruments, especially the saw blade used during bimaxillary surgery, be new or cleaned and that previously placed plates and screws be removed at an appropriate time.

## Background

Surgical ciliated cysts, first reported in 1927 by Kubo, are complications that occur after radical maxillary sinus surgery [1]. They are also known as postoperative maxillary cyst, implantation cyst, ectopic ciliated cyst, and respiratory cyst [2–4]. Most surgical ciliated cysts are found in the maxillary molar area. The reported incidence has been high in Japan, where these lesions account for 20% of oral cysts [5, 6]. They have respiratory-type epithelium lining inside, and the presumed cause is infiltration of the mucous membrane of the maxillary sinus by an instrument during surgery [6]. Surgical ciliated cysts in the mandible are very rare; only 15 cases have been reported in English-language journals so far [2–4, 7, 8]. We report a 16th case of surgical ciliated cyst in the mandible, presumed to have occurred after orthognathic surgery and genioplasty performed 24 years earlier.

## Case presentation

**Fig. 1:**
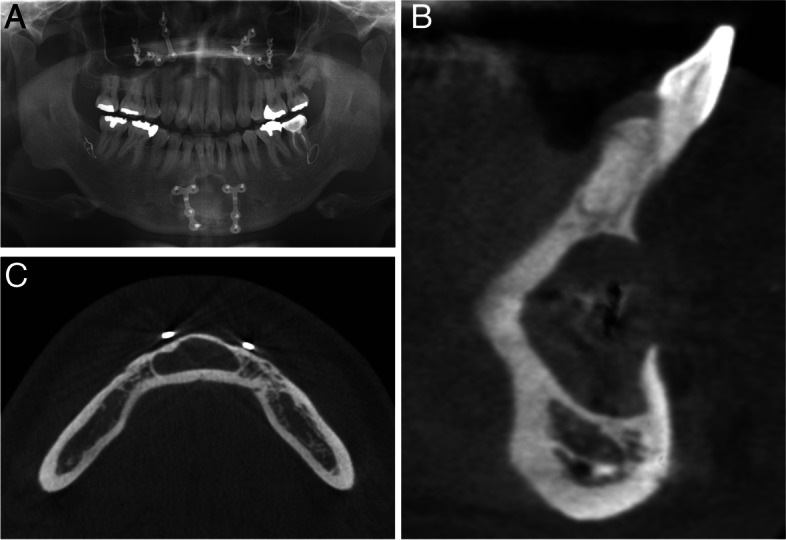
Preoperative radiographs. **A** Panoramic view. **B** Sagittal cone-beam computed tomographic (CBCT) image. **C** Axial CBCT image. A radiolucent lesion in the anterior mandible with two plates and labial cortical bone perforation were observed

A 42-year-old man was referred to the Department of Oral and Maxillofacial Surgery, Seoul National University Dental Hospital, for treatment of a cyst in the anterior mandible that was found at a local dental clinic. Cone-beam computed tomography revealed a radiolucent lesion with a diameter of > 2 cm and a labial cortical bone perforation in the lower anterior region, and the cyst was in contact with two fixed metal plates (Fig. 1). The patient stated that he had undergone orthognathic surgery and genioplasty at another hospital 24 years earlier. The patient had no medical history except for smoking half a pack a day, and he did not complain of any other symptoms such as pain or paresthesia in the affected area.

**Fig. 2:**
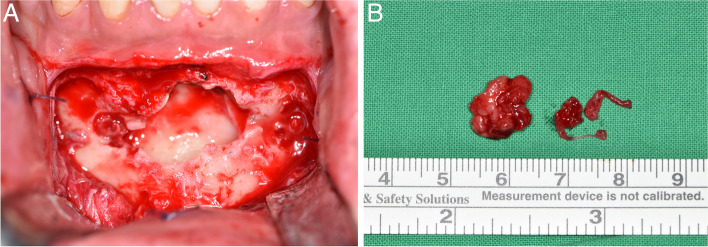
Clinical photographs during enucleation surgery. **A** and **B** The cystic mass was removed with surgical curettage after the overlying metal plates were removed

While the patient was under general anesthesia, the cyst was enucleated. A full-thickness alveolar mucosal flap was constructed via an incision in the anterior part of the mandible. The anterior part of the cyst was attached to the periosteum and was dissected and enucleated (Fig. 2). After resection of the soft tissue remaining on the cyst wall, a specimen was sent to the Department of Oral Pathology for definite diagnosis. Two four-hole plates and eight screws were removed. Primary sutures with absorbable, and nonabsorbable suture thread were placed.

**Fig. 3:**
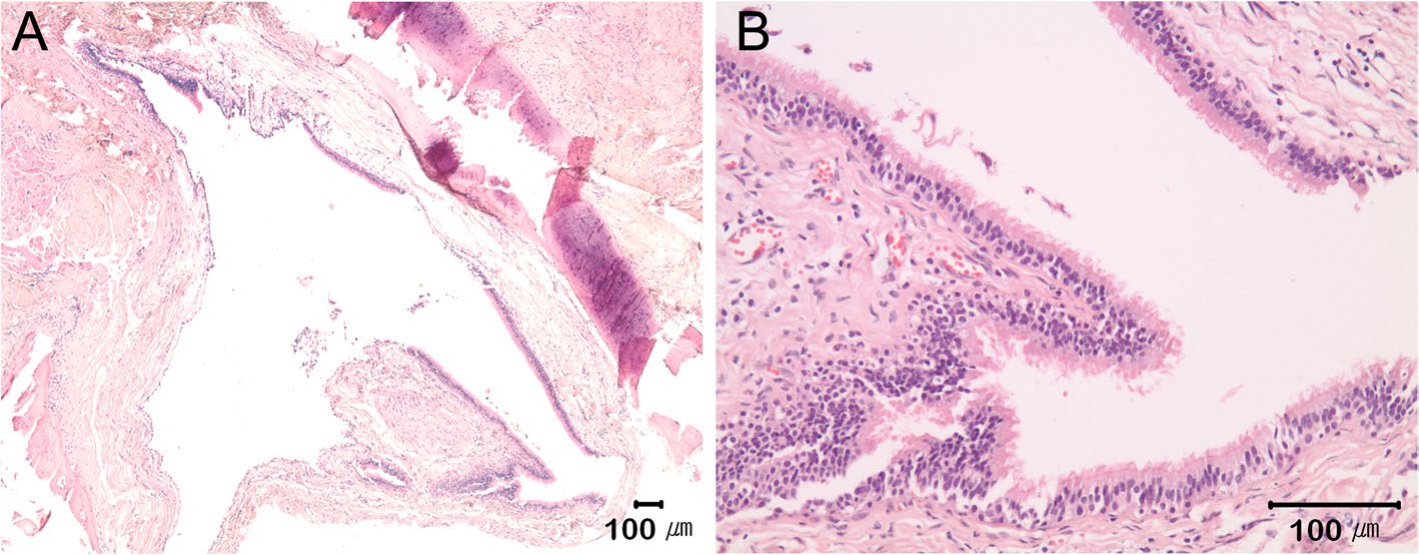
Hematoxylin and eosin staining revealed pathologic features of the cyst. The cyst wall was lined by ciliated pseudostratified columnar respiratory-type epithelium. **A** Magnification, ×40. **B** Magnification, ×100

**Fig. 4:**
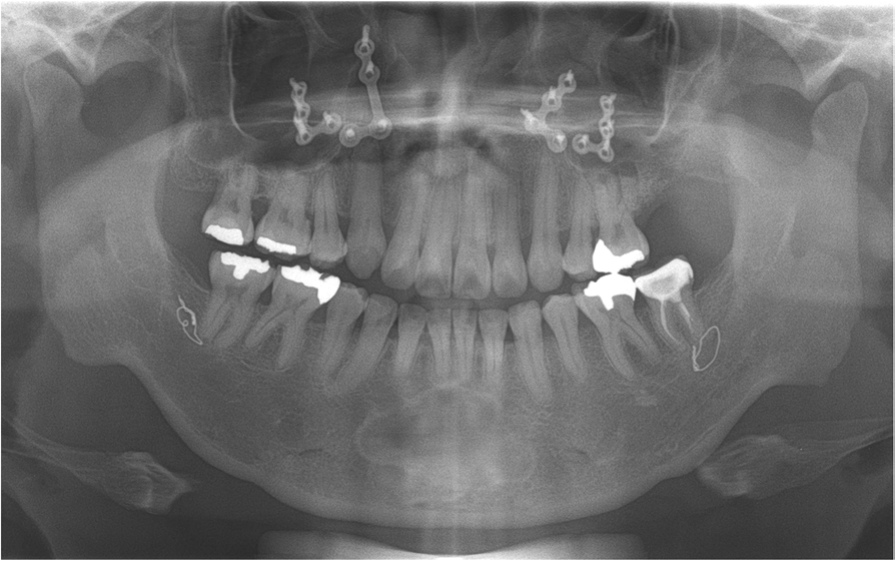
Follow-up panoramic view 7 months after surgery. The defect in the area of the cystic cavity was filled with bone that was comparable with the surrounding bone, and no sign of recurrence was observed

Histopathological examination revealed that the cyst was lined inside with ciliated respiratory epithelium, and a surgical ciliated cyst was diagnosed (Fig. 3). No recurrence was observed on panoramic radiographs 7 months after the surgery (Fig. 4).

## Discussion

Surgical ciliated cysts in the mandible are extremely rare. To the best of our knowledge, only 15 cases other than ours have been reported in English-language journals so far; they are summarized in Table 1. The lesions were discovered 2–56 years after surgery, which was presumed to be the cause of the cysts, and most affected patients had signs and symptoms such as swelling, pain, or pus discharge. Of the 16 cases, 12 occurred in the anterior part of the mandible. In all reported cases, patients had undergone multiple concomitant surgical procedures in the areas where the cysts developed. Most of the anterior mandibular cysts occurred in patients who underwent surgery in the chin region, such as chin augmentation or genioplasty accompanying a septoplasty or LeFort I osteotomy; in contrast, mandibular ramal cysts can occur after bimaxillary orthognathic surgery.

**Table 1:** Cases of mandibular surgical ciliated cyst published in English-language journals

Author/year	Age/sex	Site of lesion	Signs and symptoms	Surgical history
Nastri and Hookey (1994) [9]	33/female	Anterior mandible	Ulceration and episodic pain for 3 years and gradual swelling for 18 months	Simultaneous reduction rhinoplasty and chin augmentation with free bone and nose cartilage, 15 years earlier
Anastassov and Lee (1999) [10]	53/male	Anterior mandible	Gradual expanding of mandible and aesthetic deterioration for 6 months	Simultaneous reduction rhinoplasty and septoplasty with chin augmentation, 39 years earlier
Kelly et al. (2000) [11]	56/female	Anterior mandible	Swelling with tenderness and pain for several weeks	Simultaneous rhinoplasty and chin augmentation with nasal bone, 40 years earlier
Drmeddent and Schwartz (2001) [12]	59/male	Anterior mandible	Acute swelling of lower lip and lower labial vestibule with purulent discharge for several days	Simultaneous septorhinoplasty and chin augmentation with nasal bone and cartilage, 40 years earlier
Koutlas et al. (2002) [13]	34/female	Left mandibular ramus	Swelling and mild discomfort with intraoral fistula	Simultaneous orthognathic surgery of maxilla and mandibula, 13 years earlier
Bourgeois and Nelson (2005) [5]	27/female	Left mandibular canine and first premolar area and right mandibular incisor area	Asymptomatic, milky-white, semipurulent aspirate	LeFort I osteotomy, vertical zygomaticomaxillary osteotomy, and sliding genioplasty with submental liposuction, 4 years earlier
Lazar et al. (2006) [14]	24/male	Anterior mandible	Swelling, redness, slight discomfort, and pain for 6 weeks	Simultaneous rhinoplasty and chin augmentation with resected nasal hump, 6 years earlier
Ragsdale et al. (2009) [15]	30/male	Anterior mandible	Acute pain and swelling with purulent discharge	LeFort I osteotomy and sliding genioplasty, 16 years earlier
Li et al. (2014) [2]	72/male	Anterior mandible	Painless swelling	Simultaneous rhinoplasty and genioplasty, 56 years earlier
	42/male	Right mandibular ramus	Mental nerve paresthesia	Segmented four-piece LeFort I osteotomy, BSSO with bone graft, and genioplasty, 18 years earlier
Cai et al. (2015) [7]	23/male	Anterior mandible	Gradual swelling and tenderness	LeFort I osteotomy and genioplasty with autogenous bone graft, 28 months earlier
Seifi et al. (2016) [4]	37/female	Anterior mandible	Mild inflammation	LeFort I osteotomy and genioplasty, 2 years earlier
Syyed et al. (2018) [3]	38/male	Anterior mandible	No symptoms	LeFort I osteotomy and BSSO with augmentation genioplasty, 18 years earlier
	25/female	Anterior mandible	Swelling for 2 weeks	LeFort I osteotomy, BSSO, and genioplasty, 10 years earlier
Lafuente-Ibáñez de Mendoza et al. (2021) [8]	50/male	Posterior mandible	No symptoms	Platelet-rich plasma graft with collagen membrane on extraction socket, 2 years earlier
Our report	42/male	Anterior mandible	No symptoms	Bimaxillary orthognathic surgery and sliding genioplasty, 24 years earlier

*BSSO* Bilateral sagittal split osteotomy

The major difference between surgical ciliated cysts in the maxilla and those in the mandible is the developmental cause. Maxillary surgical ciliated cysts are hypothesized to develop either from the entrapment of sinus mucosal remnants or as a result of early closure of the maxillary ostium. The lesions can develop after sinus surgery; LeFort I, II, or III osteotomy; trauma; or traumatic extraction [16, 17]. Mandibular surgical ciliated cysts, however, are preceded by simultaneous surgery on the maxilla and mandible, such as chin augmentation with septal cartilage or bimaxillary orthognathic surgery. Of the 16 cases reported, 8 developed after nasal cartilage and bone transplantation, which supports the hypothesis that respiratory epithelium attached to the graft and transferred to the surgical site gives rise to surgical ciliated cysts [15].

Conversely, cysts developed in seven patients who had not undergone transplantation, which supports the hypothesis that the sinonasal epithelium attached to the saw blade used for maxillary osteotomy is transferred to the mandible, which gives rise to the cyst [5]. To prevent this, do not include the treatment option of bone or cartilage transplantation at the time of treatment planning, or if a cutting saw is used to cut the maxilla, a new one should be used before mandibular osteotomy [4, 5, 7]. Furthermore, to prevent the sinonasal epithelium from being transferred to the mandible by the surgical instrument, it would be advantageous to clean or replace not only the cutting saw but also other surgical instruments, such as drill bits and chisels. In only 1 of the 16 cases—the one published most recently before our report—a mandibular surgical ciliated cyst was observed after grafting of platelet-rich plasma on the extraction socket, without simultaneous maxilla and mandible surgery [8]. Those authors assumed that the cyst was caused by growth factor in the platelet-rich plasma, but this was the only such case, and more research on the causes is needed in the future.

As in our case, if the cyst is attached to the bony wall and to the soft tissue, complete removal could be difficult, and the lesion may recur. According to Soares et al., of the 17 ciliated cysts resected, none recurred [18]. In that study; however, the follow-up after removal of the lesions was short (mean: 8.6 months), and the number of samples was small; therefore, we recommend regular checkups after surgery. Also, it may be recommended that any plate and screw used for previous orthognathic surgery could be removed at an appropriate time to reduce the possibility of cyst occurrence.

In summary, surgical ciliated cysts in the mandible are extremely rare, but they could occur after simultaneous surgery on the maxilla and mandible, even decades later. For differential diagnosis, periapical cysts or odontogenic keratocysts, similar to osteolytic, should be ruled out. To prevent surgical ciliated cysts in the mandible, we recommend that the surgical instruments, especially the saw blade used during bimaxillary surgery, be new or cleaned and that previously placed plates and screws be removed at an appropriate time. Because little is currently known about recurrence of surgical ciliated cysts, we also recommend long-term follow-up after cyst enucleation.

## Data Availability

Data sharing is not applicable to this report as no data sets were generated or analyzed during the current study.

## Abbreviation

BSSO: Bilateral sagittal split osteotomy
